# Computational Exploration of Phenolic Compounds from Endophytic Fungi as α-Glucosidase Inhibitors for Diabetes Management

**DOI:** 10.1021/acsomega.4c08872

**Published:** 2024-12-19

**Authors:** Sai Anand Kannakazhi Kantari, Subbarao Kanchi, Bhargav Patnaik, Ashok Agraharam

**Affiliations:** † Department of Biosciences, Sri Sathya Sai Institute of Higher Learning, Prasanthi Nilayam, Sri Sathya Sai District, Puttaparthi, Andhra Pradesh 515134, India; ‡ Department of Physics, Sri Sathya Sai Institute of Higher Learning, Prasanthi Nilayam, Sri Sathya Sai District, Puttaparthi, Andhra Pradesh 515134, India

## Abstract

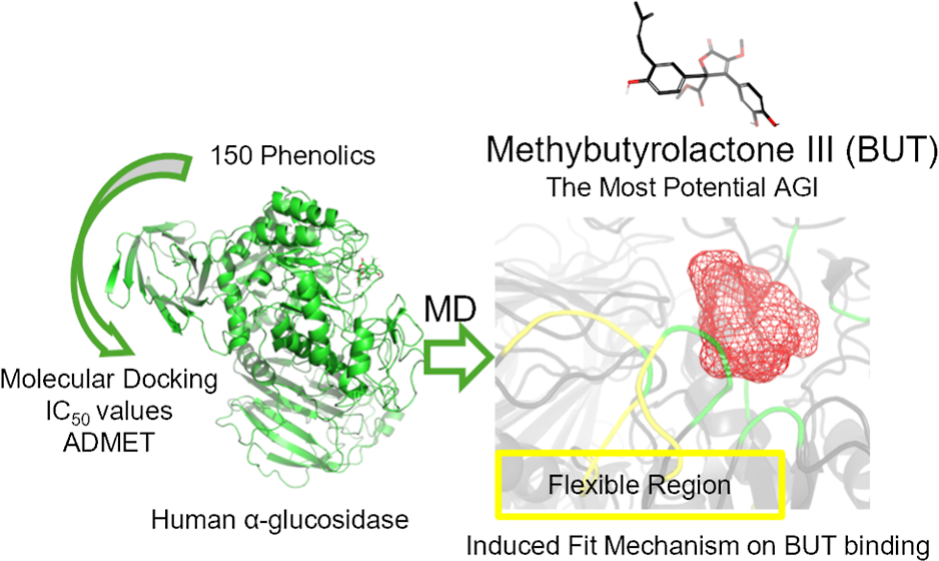

Diabetes has become a global epidemic, affecting even the younger people on an alarming scale. Inhibiting intestinal α-glucosidase is one of the key approaches to managing type 2 diabetes (T2D). In the present study, phenolic compounds (PCs) produced by endophytic fungi as potential α-glucosidase inhibitors (AGIs) are explored through ADMET profiling, molecular docking, and molecular dynamics (MD) Simulations. After 150 PCs were screened for their drug-likeness and toxicity properties, 45 molecules were selected. These were subjected to molecular docking studies against human N-terminal maltase-glucoamylase (NtMGAM). Based on binding energy and IC_50_ values, the best five PCs from different chemical classes (depsidones, phenolic acids, butenolides, furanones, and polyketides) were studied for their binding dynamics with NtMGAM employing all-atom MD simulations. Among the five ligands analyzed, the methybutyrolactone III (BUT)-NtMGAM complex exhibited significantly higher active site flexibility, indicating a conformational change in response to ligand binding. BUT interacted specifically with both key residues, Asp443 and Phe575, critical for enzyme–inhibitor stability. These interactions, coupled with increased flexibility, suggest enhanced stabilization of BUT in the active site pocket. BUT also exhibited one of the most favorable toxicity profiles among molecules analyzed using ProTox 3.0. Molecular mechanics Poisson–Boltzmann surface area calculations confirmed that BUT had the highest binding energy (−35.01 kcal/mol) driven by substantial van der Waals and electrostatic interactions. Another butenolide derivative, aspernolide (ALD) ranked second in the binding energy score (−31.13 kcal/mol). These findings suggest that PCs possessing butenolide scaffolds, like BUT and ALD, hold great promise as potential AGIs for managing T2D. These findings, however, need to be further validated through in vivo experimentation.

## 1 Introduction

Diabetes mellitus (DM) is currently considered an epidemic that has overtaken the world and is one of the most complex lifestyle disorders plaguing humanity. Type 2 diabetes (T2D) in the long run, in a majority of cases, leads to serious complications such as cardiovascular disease, neuropathy, retinopathy, nephropathy, myopathy, heightened susceptibility to skin infections, and dementia.^1^ The most disconcerting aspect today is that Type 2 DM is manifesting in persons in their late twenties or early thirties, in contrast, usually in those in their early fifties, a few decades ago.^2^ It is a well-established fact that the α-glucosidase enzyme is a significant target for therapeutic intervention in the treatment of carbohydrate-related diseases. The action of α-glucosidase occurs in the small intestine, where it facilitates the breakdown of oligosaccharides and disaccharides into monosaccharides, thereby completing the process of carbohydrate digestion. Multiple studies have shown that α-glucosidase inhibitors (AGIs) decelerate the breakdown and absorption of carbohydrates resulting in postprandial blood glucose levels hovering around the normal level.^3^ AGIs are considered to produce milder side effects compared to other oral antihyperglycemic medications since their site of action is the intestinal lumen rather than interfering with various metabolic processes going on in the body.^4^

Metformin, rosiglitazone, sulfonylurea, acarbose, and miglitol are being used extensively in the treatment of diabetes. Nevertheless, these medications negatively impact the liver and the intestine. Hence, exploring the nature of suitable alternative sources of safe antidiabetic medications is the way forward.^5^ Evaluating fungi as sources of bioactive metabolites has remained rudimentary despite their enormous potential.^6^ Endophytic fungi (EF) are an even lesser-studied group.

EF can synthesize a diverse range of bioactive metabolites, some of which exhibit pharmacological properties similar to those synthesized by plants. They cover a wide variety of metabolites belonging to distinct chemical classes, including alkaloids, terpenoids, and phenolic molecules. The polyketide pathway is the primary route of fungal biosynthesis. An important example of the use of natural polyketides in the treatment of human diseases is the path led to the discovery and development of statins.^7^

The current study specifically examines different classes of phenolic compounds (PCs) obtained from EF known for their ability to inhibit yeast α-glucosidase. These categories include polyketides, depsidones, butenolides, and benzofurans, which have an oxygen heterocycle core. Oxygen heterocycles are the second most prevalent group of heterocycles found to be part of medications approved by the U.S. Food and Drug Administration.^8^ Additional investigations are required to evaluate their mechanisms of action and potential use as AGIs. Phenolic acids, a subclass of PCs, possess resonance-stabilized structures, exhibit antioxidant properties through radical scavenging, electron donation, and singlet oxygen quenching.^9^ Depsidones are a unique group of chemicals composed of two rings of 2,4-dihydroxybenzoic acid connected via ether and ester linkages.^10^ Furanones are a group of heterocyclic chemicals that are essential components of several complex natural substances that exhibit a diverse range of biological functions. Furanone derivatives, commonly referred to as butenolides, are unsaturated γ-lactones that are present in a variety of natural products. These classes of PCs are known for exhibiting a wide variety of actions, such as anti-inflammatory, anticancer, antidiabetic, antibacterial, etc., attracting considerable attention in medical research.^11^

Computational tools utilizing AI/ML are playing a major role in small molecule research and development, accelerating the pace of traditional drug discovery efforts to a new level. Some of these include virtual screening, structure-based and ligand-based drug design, absorption, distribution, metabolism, excretion, and toxicity (ADMET) prediction, multitarget prediction, and drug repurposing. These approaches also contribute to the development of more effective, safer, and personalized medicines.^12,13^

Prediction of ADMET properties in the early stage of drug development is very crucial. While ADMET tools can reliably predict pharmacokinetic and toxicity properties, improving prediction accuracy is an ongoing effort. Despite limitations, these computational methods have yielded notable successes such as the discovery of novel opioid analgesics and SARS-CoV-2 protease inhibitors. In recent years, numerous free online web servers for predicting ADMET properties have come into existence. A detailed analysis of 18 such web servers revealed that ADMETlab stands out as the most comprehensive and accurate platform available today. ADMETlab offers the best coverage of essential pharmacokinetic parameters and provides superior precision in its predictions, making it a valuable resource for academic researchers as well as pharmaceutical companies to improve their drug discovery efforts and reduce late-stage failures.^14^

Molecular modeling techniques have become indispensable tools helping in cutting down on time and expenses, thereby accelerating the pace of drug discovery. Docking predicts how small molecules bind to protein targets, enabling virtual screening of large compound libraries and estimation of their binding affinities, despite the challenge of explaining flexibility (Flex) shown by the protein. Molecular dynamics (MD) simulations complement docking by modeling the dynamic motions of biomolecular systems, generating conformational ensembles, and assessing complex stability. Molecular modeling is poised to play an increasingly vital role in identifying and optimizing drug candidates more efficiently.^15−18^

We therefore conducted ADMET analysis, molecular docking, and MD simulation studies of 150 PCs produced by EF belonging to different classes to evaluate their potential as drug candidates for diabetes. All these molecules are reported to inhibit the yeast α-glucosidase enzyme with significant IC_50_ values. Our study aims to understand their mechanism of interaction, as well as their potential in inhibiting the human intestinal α-glucosidase enzyme (NtMGAM).

## 2 Methodology

### 2.1 Software Tools

MarvinSketch 6.3, Molsoft L.L.C. 3.9, ProTox 3.0, ADMETlab 3.0, Modeler 12.0, UCSF Chimera-1.17.3, PyRx V 0.9, Auto Dock Vina 1.2.0, Open Babel 3.2.0, Protein Data Bank (PDB), PubChem, PyMOL 2.5, COACH-D 2.0, LigPlot+ 2.2, and GROMACS Version 5.

### 2.2 ADMET Analysis

Phenolics encompass a wide spectrum of substances, ranging from a basic, small-sized molecule with a single aromatic ring to highly intricate structures such as depsidones, butenolides, and isocoumarins. PCs are well-known for their beneficial health effects, as well as lesser toxicity. Most of the phenolic molecules are potent antioxidants with high free radical scavenging ability.^19,20^ 150 PCs with different scaffolds produced by EF, known for their ability to inhibit α-glucosidase activity, were evaluated for their potential as antidiabetic drug.^21^ A comprehensive ADMET analysis was employed to evaluate the pharmacokinetic properties of these PCS.

Ligand structures were either retrieved from the PubChem database or generated using MarvinSketch software. The simplified molecular input line entry system (SMILES) notation of the 150 PCs was generated and input into the drug-likeness (DL) prediction tool, Molsoft L.L.C. 3.9. The Molsoft program utilizes a comprehensive set of molecular descriptors to evaluate the DL score (DLS) of molecules. These descriptors include molecular weight (MW), number of hydrogen bond donors and acceptors, lipophilicity (octanol–water partition coefficient, or logP), topological polar surface area (TPSA), molecular volume, aqueous solubility (logS), blood–brain barrier score, p*K*_a_ of the most basic/acidic group, number of stereocenters, and rotatable bonds. DLS is a parameter that estimates how well a compound aligns with the properties typically observed in drug molecules. By calculating these various physicochemical and structural properties Molsoft’s algorithm generates a DL prediction score.^22−24^ PCs with a DLS of ≥0.18 were selected for further analysis. The cutoff of 0.18 is significant because it serves as a threshold to distinguish between drug-like and nondrug-like molecules. Molecules with a DLS above 0.18 are more likely to possess properties consistent with those of known drugs, suggesting that they have a higher potential to be accepted as drugs. Conversely, molecules scoring below this threshold may not exhibit desirable drug-like characteristics.^25,26^

ProTox is an advanced in silico toxicity prediction platform that has evolved through several versions, including ProTox-II and the latest ProTox-3.0.^27^ It predicts various toxicity end points for molecules such as acute toxicity, hepatotoxicity, cytotoxicity, carcinogenicity, mutagenicity, and immunotoxicity, using a combination of computational techniques including molecular similarity analysis, pharmacophore modeling, fragment propensity analysis, and machine learning algorithms. ProTox categorizes molecules into toxicity classes based on their predicted LD50 values, with Class I being highly toxic (≤50 mg/kg) and Class VI being relatively harmless (>15,000 mg/kg). Molecules classified as Class 4 or above (including Classes 4, 5, and 6) are considered to have lower toxicity levels, with Class 4 being slightly toxic (5000–15,000 mg/kg), Class 5 practically nontoxic (>15,000 mg/kg), and Class 6 relatively harmless (>15,000 mg/kg).^28−31^ ProTox 3.0 was employed to predict the toxicity profiles of PCs with DLS ≥ 0.18, providing insights into their potential adverse effects. Metabolites falling into the toxicity Class 4 or above were selected for further study.

**Figure 1:**
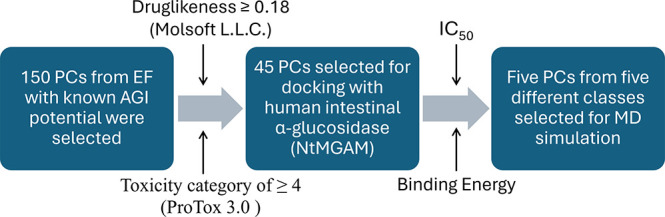
Overview of the methodological process.

The top 45 PC candidates with druglike properties and low toxicity were selected for docking analysis. ADMETlab 3.0 web server was used to predict the ADMET properties of the selected phenolics, with a specific focus on knowing about their physicochemical properties and toxicity profiles.^32^ Based on the docking scores and IC_50_ values, 5 PCs were selected for MD simulation analysis (Figure 1).

### 2.3 Molecular Docking

Molecular docking was employed to study the interactions between 45 PCs from EF and Nt-MGAM, which cleaves shorter chain oligosaccharides. These 45 phenolics were selected from an initial pool of 150 based on their DL and toxicity analysis.

Crystal structure of Nt-MGAM (2QMJ) was obtained from the RSCB PDB. All heteroatoms’ atoms, including residues with attached sugar molecules, were eliminated. Missing residues the target protein were modeled using Modeler in conjunction with UCSF Chimera.^33^ Molecular docking analysis was carried out utilizing PyRx virtual screening software (V 0.9) which operates using the Auto Dock Vina configuration.^34^ Active site residues were predicted using the COACH-D web server and supported with findings from the literature.^35,36^ Docking process utilized grid-specific rigid docking within a box with dimensions *X*: 25.8199, *Y*: 22.8033, and *Z*: 24.6510, which was centered around the active site residues. Acarbose, a well-known α-glucosidase inhibitor, was added as a control for virtual screening. Nine poses were created for each ligand, and the one with the lowest binding energy was chosen. LigPlot + software was employed to understand the protein–ligand interactions.^37^

### 2.4 MD Simulation

GROMACS Version 5 software was employed to execute all-atom MD simulation studies.^38^ 300 ns production MD was applied to respective docked protein–ligand complexes to obtain thermodynamically stable structures. Amber99SB-ILDN force field^39^ was used for the protein, while Antechamber was employed to parametrize the ligands using the general AMBER force field parameters.^40^ The system was solvated in a box of SPC/E water molecules with a minimum distance of 1.0 nm between the solute and box edges.^41^ Counterions were added to neutralize the system. To maintain a physiological concentration of 0.15 M, Na^+^ and Cl^–^ ions were added. Energy minimization was conducted using the steepest descent algorithm to eliminate any unfavorable contacts. The system was equilibrated in two phases starting with 0.5 ns *NVT* equilibration (with position restraints on the protein at a force constant of 1000 kcal mol^–1^ Å^–2^) followed by 0.5 ns NPT equilibration. Production MD was run for 300 ns using a 2 fs time step with temperature maintained at 300 K using Berendsen thermostat and pressure maintained at 1 bar using a Parrinello–Rahman barostat.^42,43^ Periodic boundary conditions were applied in all three directions (*x*, *y*, *z*) and long-range electrostatics were treated using Particle–Mesh–Ewald method.^44^ The same protocol was followed by others earlier to obtain thermodynamically stable trajectories for systems involving both isolated proteins as well as protein–membrane complexes.^45−47^ Trajectories were analyzed for root mean square deviation (RMSD), RMSD per residue (per residue), root mean square fluctuation (RMSF), hydrogen bonds, and protein–ligand contacts using GROMACS analysis tools.^48^ RMSD per residue and close contacts were calculated using the last 50 ns trajectory. LigPlot+ was used to study the protein–ligand interactions.

### 2.5 Binding Energy Calculations

To understand protein–ligand interactions in terms of their binding characteristics, binding energy calculations were performed using the molecular mechanics-Poisson–Boltzmann surface area (MM-PBSA) method.^49^ The MM/PBSA approach has emerged as a widely used and efficient method for calculating binding free energies in molecular recognition processes, particularly for protein–ligand interactions. MM/PBSA combines molecular mechanics energies with implicit solvent models to estimate binding affinities more accurately than simple docking scoring functions.^50,51^ The last 10 ns MD trajectories were considered for MM-PBSA calculations. The molecular, mechanical, polar, and apolar solvation energies were used to evaluate the Δ*G*. Binding energy is calculated using eqs 1 and 2.
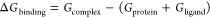
1
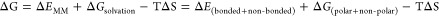
2where *G*_binding_ is the binding free energy, *G*_complex_ is the total free energy of the protein–ligand complex, *G*_protein_ and *G*_ligand_ are the total free energies of the isolated protein and ligand in solvent, respectively, Δ*G* is the standard free energy, Δ*E*_MM_ is the average molecular mechanics potential energy in vacuum, Δ*G*_Solvation_ is the solvation energy, Δ*E* is the total energy of bonded as well as nonbonded interactions, Δ*S* is the change in entropy of the system upon ligand binding, and *T* is the temperature in Kelvin.^52^

## 3 Results and Discussion

### 3.1 ADMET Analysis and Molecular Docking

Our study focused on evaluating the DL and toxicity profiles of 150 PCs and their suitability for being considered for drug development. Among these 150 PCs, 47 (31.33%) exhibited a DLS of ≥0.18. Out of these 47 high-potential PCs, 8 (17.02%) were classified as practically nontoxic (class 5), 37 (78.72%) were classified as slightly toxic (class 4), and 2 (4.26%) were classified as moderately toxic (class 3). 45 belonging to class 4 or above were selected for further investigation (Supporting Information S1).

Both the IC_50_ value and binding energy obtained from molecular docking studies are important parameters for understanding α-glucosidase inhibition by the selected PCs. Together, they provide complementary information. While IC_50_ quantifies inhibitory potency under defined conditions, docking energies and poses suggest the molecular basis for that inhibition. Molecular docking studies with NtMGAM (PDB ID: 2QMJ) were conducted on the 45 selected molecules with known IC_50_ values from diverse classes, including depsidones, phenolic acids, butenolides, furanones, and polyketides. 28 molecules exhibited binding affinities greater than or equal to the standard drug acarbose (−6.6 kcal/mol), with binding energies ranging from −5.3 to −9 kcal/mol (Supporting Information S2). From the depsidone class, botryorhodine H (BOT) was selected for MD simulation due to its superior IC_50_ (8 μM), despite having a binding energy (−6.6 kcal/mol) lower than that of colletotric A (−7.4 kcal/mol). Among phenolic acids, (*R*)-ethyl 3,5-dihydroxy-7-(8-hydroxynonyl) benzoate (BEN) showed better binding energy (−7 kcal/mol) compared to acarbose and a significant IC_50_ value (22.3 μM). It was selected for the MD simulation. Sixteen butenolide derivatives demonstrated binding energies higher than those of acarbose. While (*R*,*E*)-3-(2,2-dimethylchroman-6-yl)-4-hydroxy-5-((2-(2-hydroxypropan-2-yl)-2,3-dihydrobenzofuran-5-yl)methylene)furan-2(5*H*)-one exhibited the highest binding energy at −8.1 kcal/mol, methybutyrolactone III (BUT) with a binding energy of −7.6 kcal/mol was selected for MD due to its significant IC_50_ value (16 μM). All polyketide molecules surpassed acarbose’s binding energy, with pinazaphilones B showing the highest affinity (−9 kcal/mol) among all groups. However, aspergifuranone (ASG) was chosen for MD simulations due to its superior IC_50_ (9.05 μM) and high binding energy (−8.9 kcal/mol). From the furanones and benzofuranones classes, five molecules outperformed the standard drug, acarbose, with butyrolactone I exhibiting the highest binding energy (−8.1 kcal/mol). Aspernolide D (ALD) with a binding energy of −7.2 kcal/mol was selected for MD simulation as it was identified as one of the safest molecules in the ADMET analysis.

**Figure 2:**
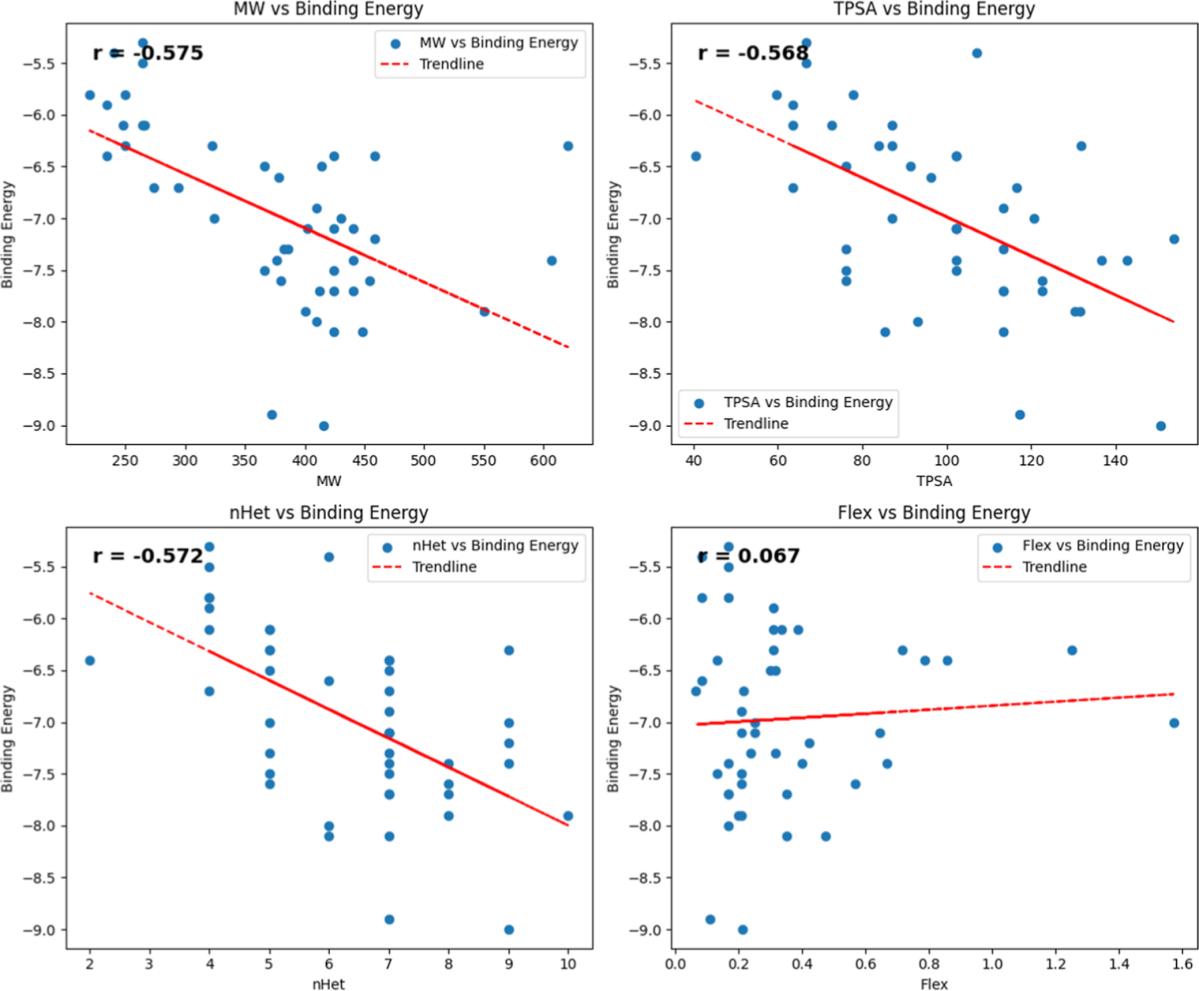
Correlation between key physicochemical properties of the 45 PCs and their binding energy with NtMGAM. There is a positive correlation between MW, TPSA, and nHets of the PCs to the binding energy, whereas flexibility did not have any correlation to the binding energy.

Physicochemical properties and toxicity profiles for the selected 45 PCs were obtained using ADMETlab 3.0. A correlation analysis was conducted to understand the relationship between key physicochemical properties [MW, TPSA, number of heteroatoms (nHet), and Flex], and binding energies of the 45 selected PCs. The results indicated a positive correlation between binding energy and MW, TPSA, and nHet, suggesting that larger, more polar and heteroatom-rich molecules tend to bind more efficiently with NtMGAM. Conversely, Flex did not show any correlation with the binding energies of the ligands (Figure 2 and Supporting Information S3).

Furthermore, a comparative analysis of the toxicity profiles of the 45 selected PCs was carried out, which included assessment for human ether-à-go-go-related Gene inhibition, drug-induced liver injury, Ame’s test score, carcinogenicity, respiratory toxicity, human hepatotoxicity, and neurotoxicity. Based on this analysis, ALD and BEN were identified to be the safest molecules among the 45 selected PCs (Supporting Information S4 and S5).

### 3.2 MD Simulation Analysis

**Figure 3:**
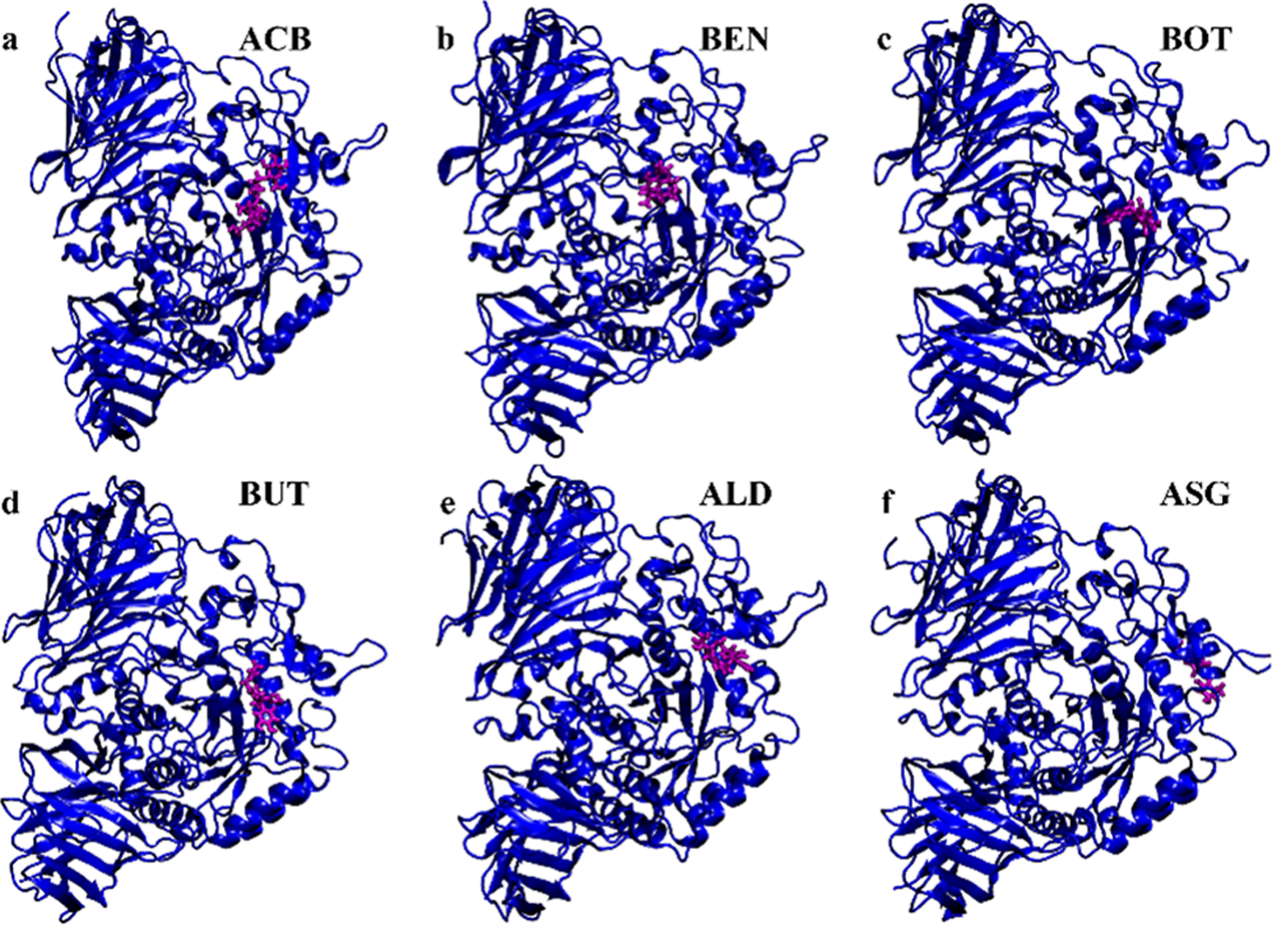
Final poses of wild-type protein with various ligands after 300 ns MD simulations. The protein structure is shown in blue, while ligands are represented in magenta. The panels represent (a) WT-ACB, (b) WT-BEN, (c) WT-BOT, (d) WT-BUT, (e) WT-ALD, and (f) WT-ASG.

Human intestinal α-glucosidase (NtMGAM) is an important therapeutic target for treating T2D and obesity. NtMGAM active site consists primarily of C-terminal β-strand residues, which are part of the (β/α)_8_ barrel motif, extending from positions 270 to 651. The structure of the substrate binding site is influenced by three key regions present at the entrance of the (β,α)_8_ barrel motif. These are (i) loop from the N-terminal domain (residues 200–217); (ii) section of catalytic Insert 1 (residues 367–416); and (iii) section of Insert 2 (residues 447–492). Flexibility of these regions determine the conformation of the substrate binding site.^36^ Five PCs (BOT, BEN, BUT, ALD, and ASG) were selected from a group of 45 molecules based on criteria such as binding energy, IC_50_ value, and ADMET properties. Each chosen compound represented a distinct chemical class: depsidones (BOT), phenolic acid (BEN), butenolides (BUT), furanones (ALD), and polyketides (ASG). The binding dynamics of these PCs with NtMGAM were investigated through MD simulation studies. During a 300 ns simulation period, the ligands exhibited a dynamic behavior within the catalytic domain, as observed in Figure 3.

#### 3.2.1 Stability and Conformational Changes

**Figure 4:**
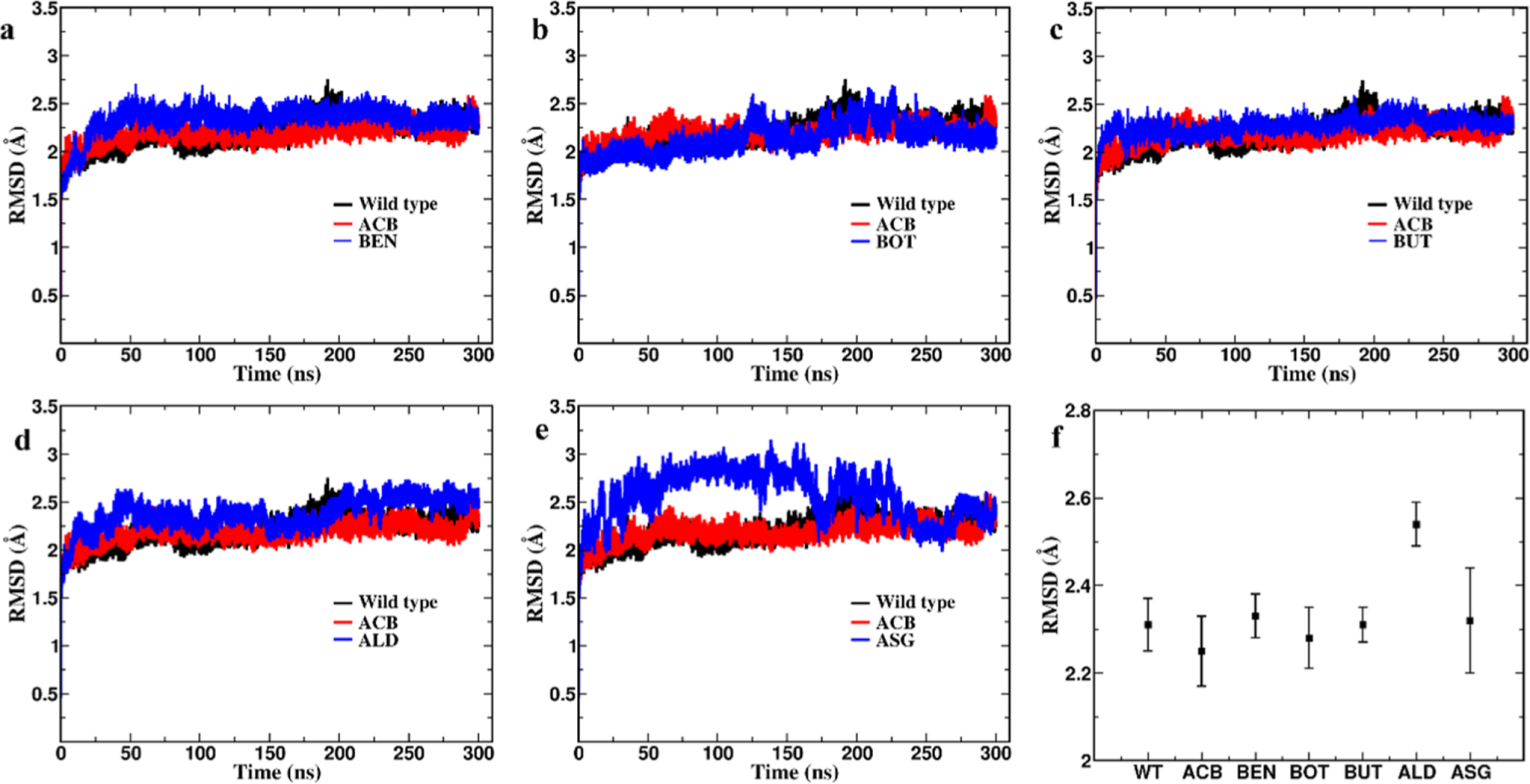
RMSD of protein–ligand complexes. Each subplot (a–e) shows the RMSD for a different ligand interacting with the protein, compared to the WT protein and standard Acarbose. The final subplot (f) summarizes the average RMSD values for each complex. The protein ligand complex reached a stable confirmation at the end of 300 ns. Due to the unbinding event RMSD of the 2QMJ–ASG complex had higher error bar. ALD has a slightly higher RMSD due to the flexibility of the N terminal chain.

**Figure 5:**
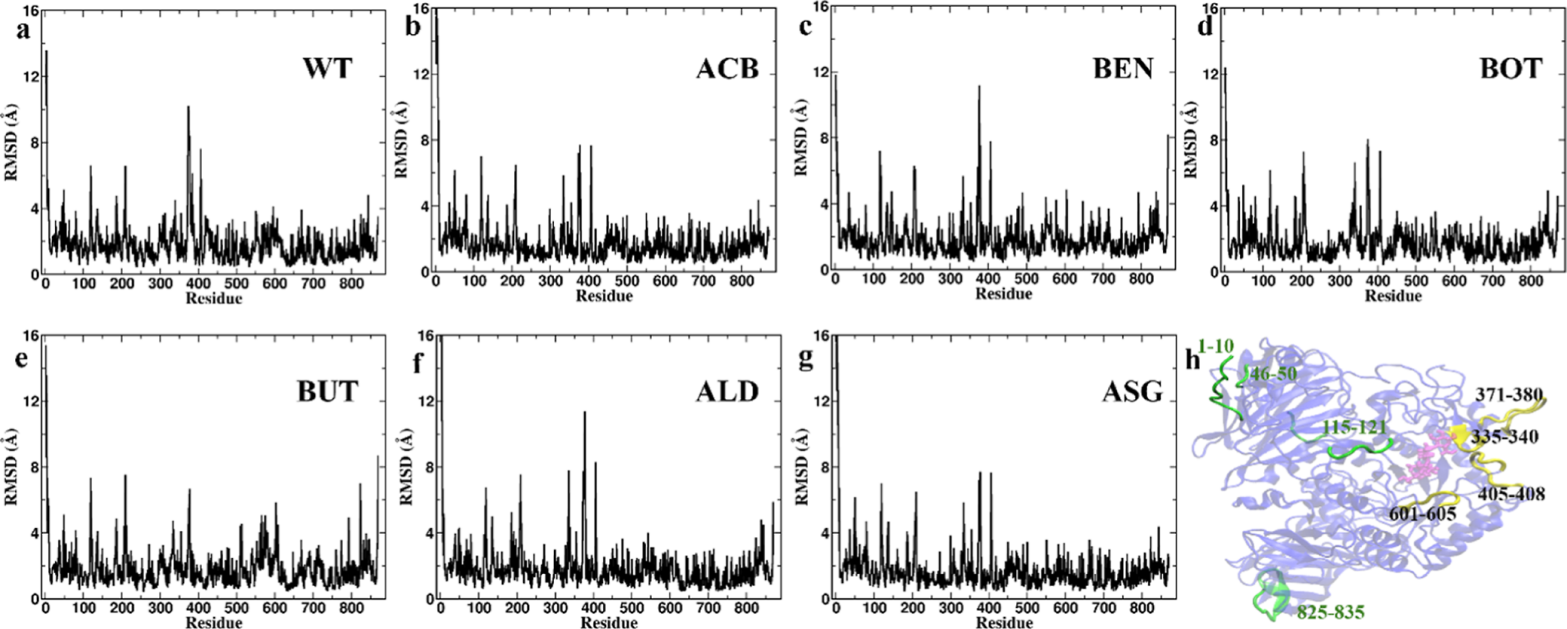
RMSD per residue analysis for protein–ligand complexes during MD simulations. Panels (a–g) display the RMSD values for each residue of the protein in the presence of different ligands: (a) WT (wild type), (b) ACB, (c) BEN, (d) BOT, (e) BUT (f) ALD, and (g) ASG. The RMSD values, measured in Angstroms (Å), indicate the deviation of each residue from its initial position over the course of the simulation. (h) Structural representation of the protein, highlighting specific residues with notable fluctuations. In the catalytic domain, the flexibility of inserted loop 1 (367–416) is observed in all the 6 cases even though the intensity is different whereas the significant flexibility above 4 Å of the active site residues 601–605 was observed only in the case of BUT suggesting changes in active site confirmation to fit the ligand.

RMSD analysis revealed that all protein–ligand complexes achieved conformational stability by the end of the 300 ns MD simulation (Figure 4). The free protein wild-type (WT), WT–BEN, WT–BOT, and WT–BUT complexes exhibited significant fluctuations until approximately 200 ns, stabilizing between 200 and 300 ns with RMSD values settling at 2.3 Å. The WT–ALD complex demonstrated a slightly higher RMSD of 2.5 Å, attributed to the flexible N-terminal end, where residues 1–10 showed an RMSD of 25 Å (Figure 5f). The WT–ASG complex displayed higher variability until 250 ns, after which it stabilized. By 300 ns, all complexes maintained stable RMSD values, suggesting consistent structural integrity and interaction stability.

#### 3.2.2 Dynamic Flexibility of Binding Regions

**Figure 6:**
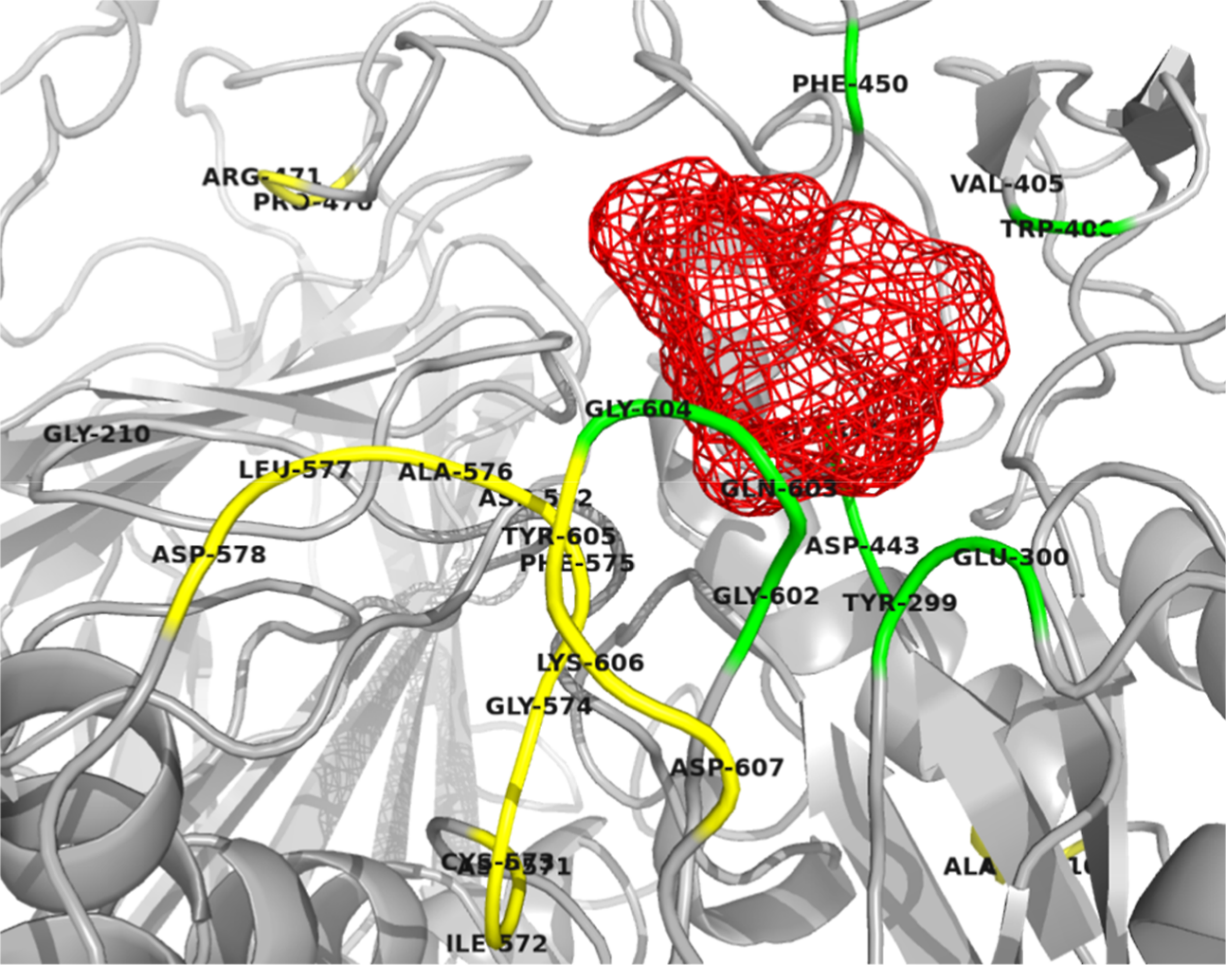
Interaction of BUT with the active site of NtMGAM was visualized using PyMOL. In the visualization, residues involved in the interaction with BUT are highlighted in green, while those exhibiting significant fluctuations are colored yellow. A significant change in the protein conformation in the active site region was observed exclusively in the presence of BUT (red color) facilitating its stable integration within the binding site.

**Figure 7:**
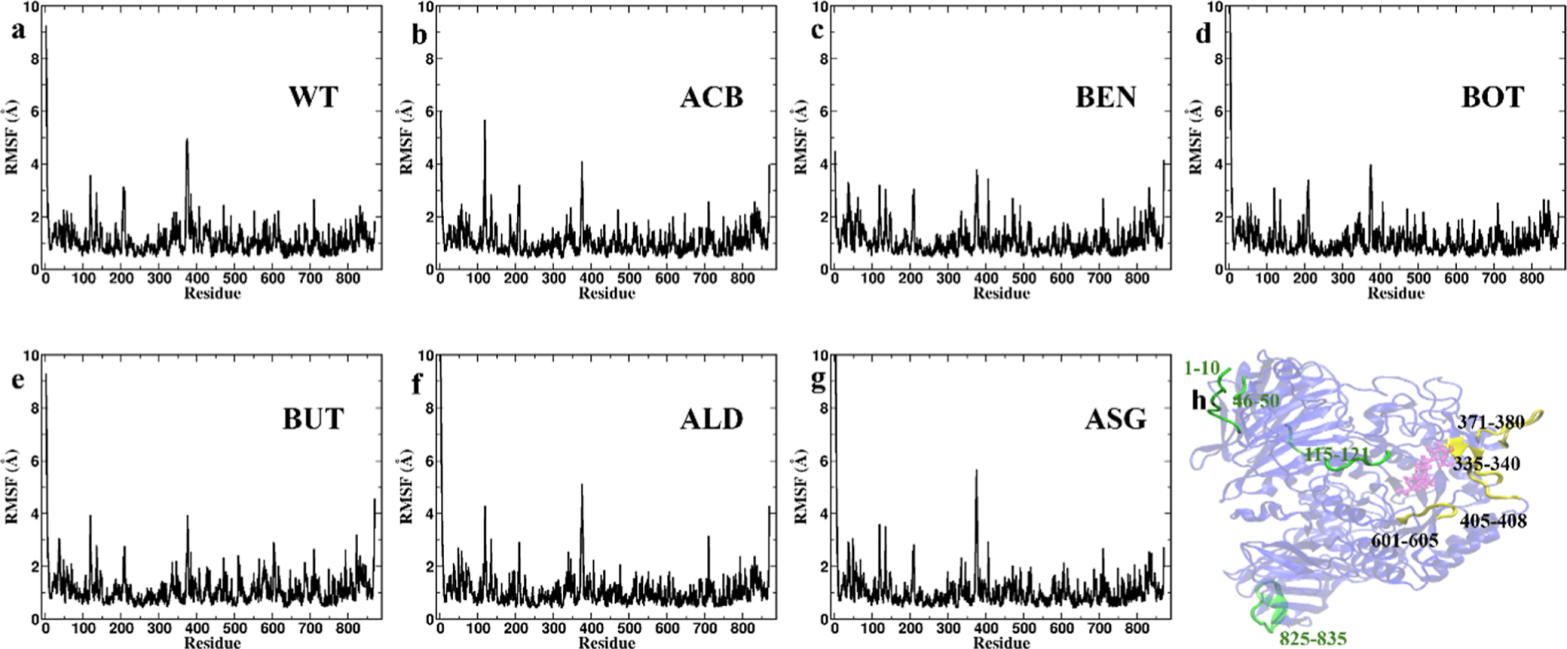
This figure illustrates the RMSFs of a protein–ligand complex averaged over last 10 ns MD simulation. The RMSF values, measured in Angstroms (Å), represent the flexibility of each residue in the protein structure. RMSF profiles for different ligands: (a) WT (wild type), (b) ACB, (c) BEN, (d) BOT, (e) BUT, (f) ALD, and (g) ASG. (h) Visual representation of the protein structure, highlighting specific residues with notable RMSF values. The RMSF values were consistent with the RMSD per residue. The flexibility residues 602–607 can be observed in 2QMJ-BUT which enhances the ligand binding with the catalytic residues.

RMSD per residue analysis (Figure 5) highlighted regions of the protein exhibiting higher flexibility or stability in response to ligand binding. Significant fluctuations (>4 Å) were observed for all six complexes in the following regions: residues 1–10, 46–50, 115–121, 371–380, and 825–835. Additionally, the following specific fluctuations were noted in the respective complexes: residues 335–340 in WT–ALD, WT–BOT, and WT–ASG; residues 405–408 in WT–ALD, WT–BOT, WT–BEN, and WT–ASG. Lesser fluctuations in region 405–408 of the WT–ACB and WT–BUT complexes suggest that their interactions stabilized the bulky hydrophobic residue Trp406, the key residue of the inserted loop 1.^36^ Residues 601–605, along with residues 565, 574, 577, and 581, which are part of the active site, exhibited significant fluctuations in the case of the WT-BUT complex, indicating conformational changes occurring to accommodate BUT within the active site (Figure 6). RMSF analysis (Figure 7) supports findings from RMSD per residue analysis, highlighting the stabilization of residues Phe405 and Trp406 in both WT–BUT and WT–ACB. Additionally, it demonstrates greater flexibility of regions 571–578 and 602–607, observed exclusively in WT–BUT. This is the first report of an inhibitor binding to the active site of human α-glucosidase, bringing about a conformational change and thereby conferring stability to the enzyme–inhibitor complex.

#### 3.2.3 Protein Ligand Interactions

**Figure 8:**
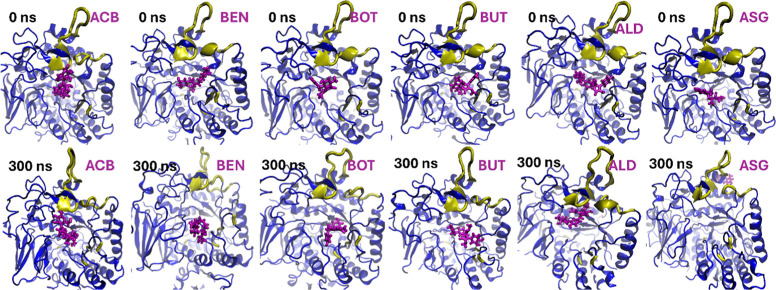
Comparison of Ligand Positions at 0 and 300 ns in MD Simulations. ACB and BUT remained in the same place showing its stability throughout the simulation. BEN and BOT showed a change in their confirmation. ALD moved toward the inserted loop and ASG moved out of the active site and bind outside.

Protein–ligand interactions at 0 and 300 ns, respectively, were compared (Figure 8). Two ligands, ACB and BUT, exhibited the maximum extended state as well as the minimum movement within the active site from 0 to 300 ns, indicating their strong and stable binding. In contrast, ligand ALD moved toward inserted loop 1, suggesting a different binding orientation, whereas BEN and BOT adopted a bent state, indicating a conformational change to maintain binding. Notably, ligand ASG exhibited maximum mobility, moving out of the active site and binding to a different region of the protein, suggesting its poor affinity for interacting with the latter.

**Figure 9:**
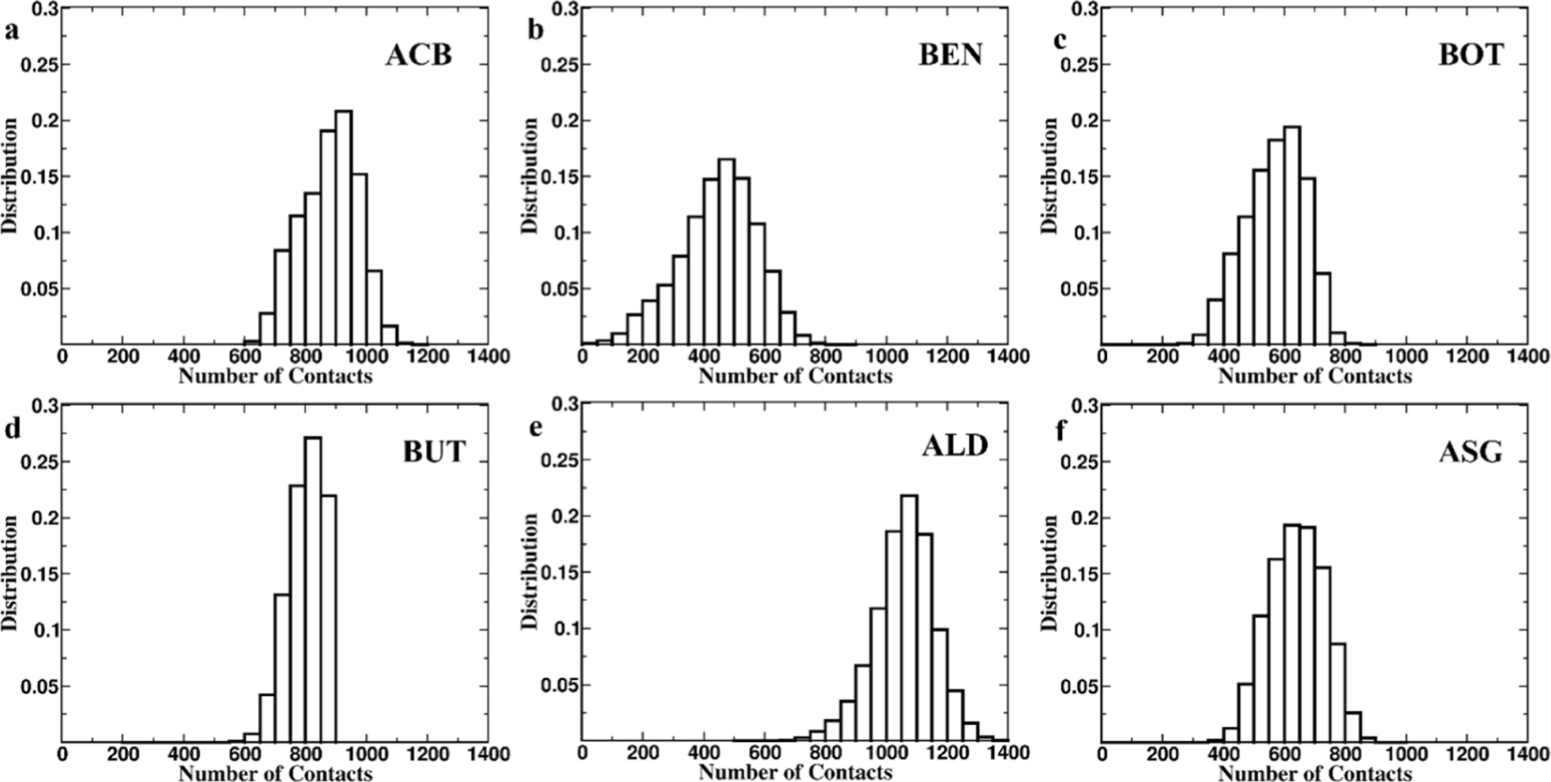
Number of Close Contacts Distribution of the number of close contacts in protein–ligand complexes during a 300 ns MD simulation. Each subplot represents a different complex: (a) ACB, (b) BEN, (c) BOT, (d) BUT (e) ALD, and (f) ASG. The *x*-axis denotes the number of contacts, while the *y*-axis shows the distribution frequency. The histograms illustrate the variations in contact distribution, reflecting the unique interaction characteristics of each complex during the simulation. ALD, ACB, and BUT has maximum contacts with the residues, and BEN has the least. BUT has the maximum stable interaction throughout the simulation.

**Figure 10:**
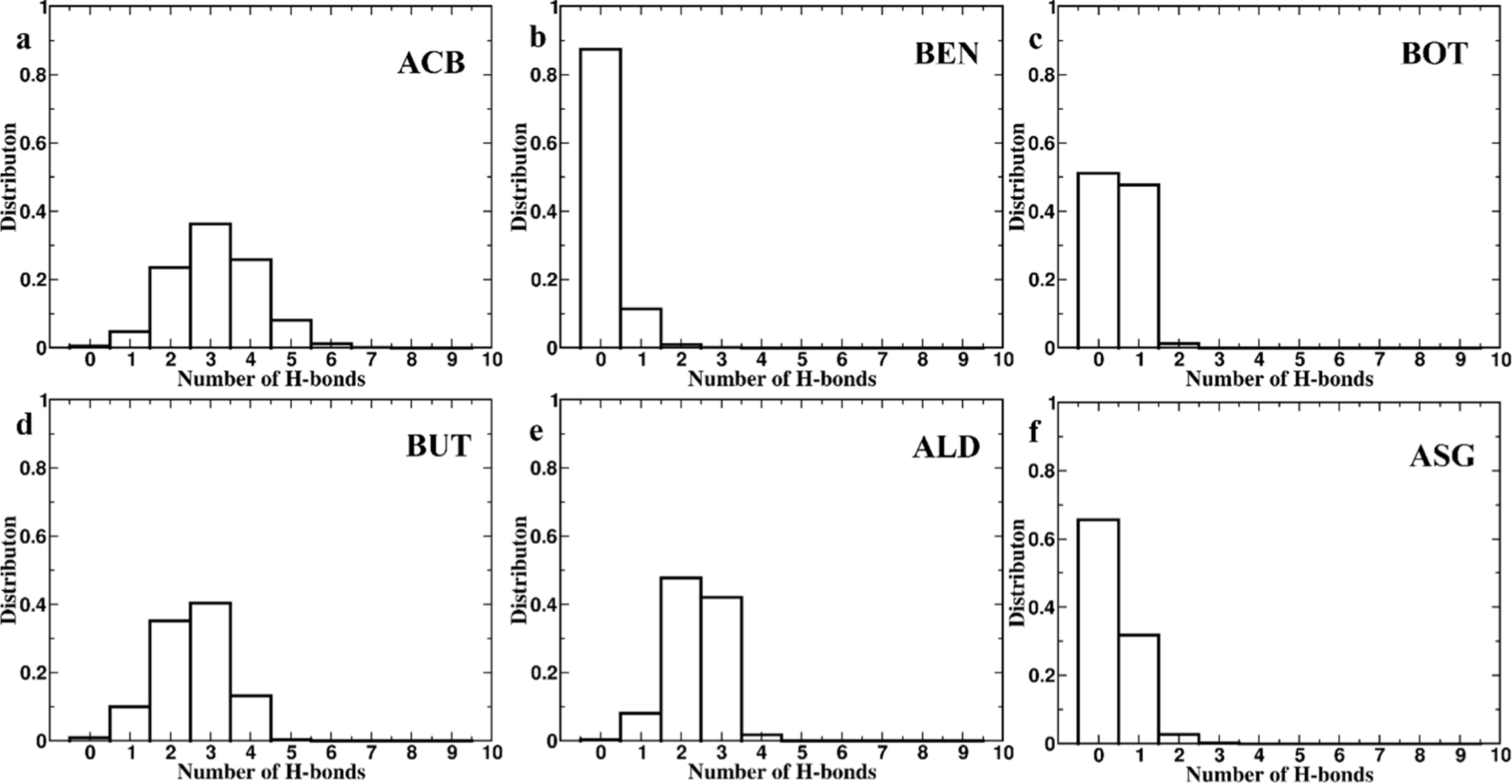
Number of hydrogen bonding distribution of the number of hydrogen bonds in 300 ns MD simulations of protein–ligand complexes. The histograms represent the frequency of different numbers of hydrogen bonds observed throughout the simulation for each complex: (a) ACB, (b) BEN, (c) BOT, (d) BUT, (e) ALD, and (f) ASG. ACB, BUT, and ALD mostly has three hydrogen bonds, whereas BEN, BOT, and ASG has either zero or one hydrogen bond interactions.

Analysis of close contacts and hydrogen bonding between the protein and respective ligands provided deeper insights into their interactions (Figures 9 and 10). The number of close contacts was highest for ALD (1100), followed by ACB (900) and BUT (800); moderate for BOT and ASG (600 each); and lowest for BEN (500). BUT displayed the narrowest range in terms of the number of close contacts throughout the period of simulation, highlighting consistency in its stability within the active site. ACB and BUT showed the highest average number of hydrogen bonds at three, followed by ALD at two and BOT, ASG, and BEN at zero (Figure 10). Based on their strong interactions with the protein, in terms of the number of close contacts as well as hydrogen bonds, ACB, BUT, and ALD seem well qualified to be potential AGIs.

**Figure 11:**
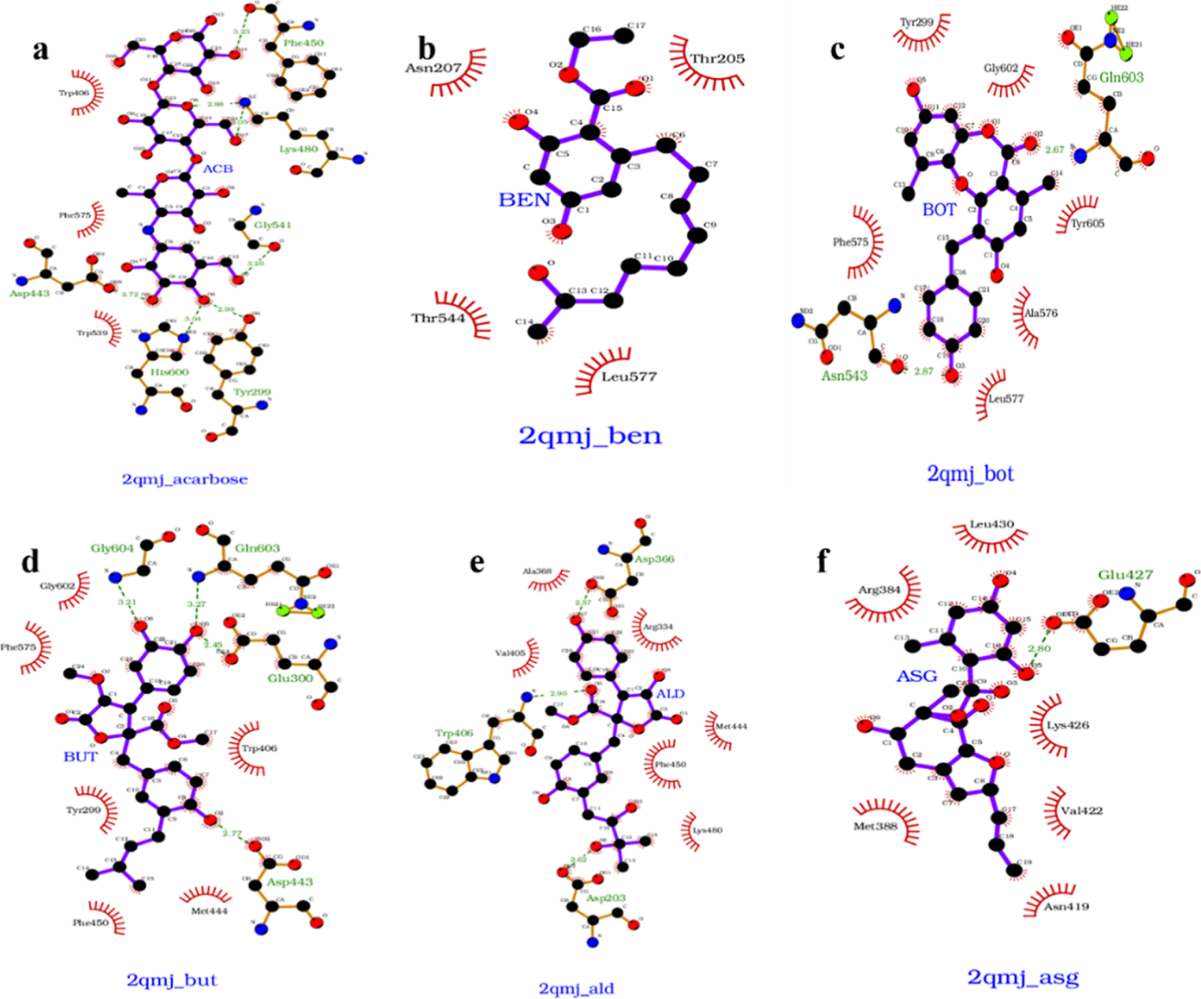
LigPlot + diagrams depicting the interactions between the protein and each ligand over a 300 ns MD simulation. Each subplot represents a different ligand: (a) ACB, (b) BEN, (c) BOT, (d) BUT, (e) ALD, and (f) ASG. Hydrogen bonds are shown as dashed lines, and hydrophobic interactions are represented by spoked arcs. ACB, ALD, and BUT have maximum interactions, whereas BEN has the least interactions. ASG does not have any interactions with the active site residues.

Ligplot analysis of the six ligands BOT, BEN, BUT, ASG, ALD, and ACB highlights several key details of their interactions with the target protein (Figure 11 and Table 1). The hydrophobic interactions and H bonding interactions within the active site play a vital role in determining the strength of binding and stability of the enzyme–inhibitor complex. Interaction with maximum number of residues were observed in BUT (10) followed ACB and ALD (9 each). ACB and BUT interacted with the catalytic nucleophile Asp443, which is a key residue for inhibitors like de-O-sulfonated kotalanol (DSK) and Maysin (MAY).^53,54^ ACB, BOT, and BUT interacted with Phe575 and Tyr299, key residues for enzyme–inhibitor stability.^55−57^ Gln603 formed hydrogen bonds with BOT and BUT. Similar interaction was reported involving another inhibitor, L2.^57^ ALD formed a hydrogen bond with a catalytic residue, Asp203.^58^ ASG interacted with a unique set of residues (Glu427, Arg384, Met388, Asn419, Val422, Lys426, and Leu430) that are not part of active site, indicating a different binding location for it. Interestingly, BEN unlike ACB, BUT, and ALD, formed no hydrogen bonds with active site residues. BEN relied solely on hydrophobic interactions for binding, engaging four residues (Thr205, Asn207, Thr544, and Leu577) located at the periphery of the active site. This suggests that BEN binds in a region distinct from the NtMGAM’s primary catalytic site.

**Table 1:** Details of Protein–Ligand Interaction after 300 ns MD Simulation, Including Ligand Name and Class, No. of H Bonding Interactions, No. of hydrophobic interactions, Residues Forming Hydrogen Bonds, and Total Interaction with Active Site Residues (NASR)

s.n.	LIGS	class	NHB	NHI	H-bond residues	NASR
1	ACB	Pseudo-TS	6	3	Tyr299, Asp443, Phe450, Lys480, Gly541, His600	4
2	BEN	phenolic acid	0	4	none	1
3	BOT	depsidone	2	6	Asn543, Gln603	2
4	BUT	butenolides	4	6	Glu300, Asp443, Gln603, Gly604	4
5	ALD	butenolides	3	6	Asp203, Asp366, Trp406	3
6	ASG	polyketide	1	6	Glu427	0

The catalytic domain of NtMGAM (residues 270–651) includes two inserted loops (367–416 and 447–492, respectively), which influence the active site pocket. Trp406, part of the first inserted loop, is the key for inhibitor stability as well as for its stacking interactions with sugar rings.^55−57,59^ It is important to note that Trp406 was involved in interactions with BUT, ALD, and ACB. Met444 and Phe450, which are part of the second inserted loop region, were involved in interactions with BUT and ALD. They are important for stabilizing the ligand.^60^ These observations suggest that residues from both the inserted loop regions play a significant role in ligand binding, particularly in the case of BUT, ALD, and ACB. ALD binding to multiple residues (Trp406, Arg334, Ala368, Val405, Met444, Phe450, and Lys480) of both the inserted loops simultaneously could be preventing entry of the substrate into the active site pocket, thereby making it a good inhibitor candidate.

Fluctuation of Trp406, a key residue in the first inserted loop (367–416), is associated with pocket widening and premature ligand dissociation observed with DSK.^59^ RMSF and RMSD per residue analyses revealed that in ACB and BUT complexes Trp406 maintained a high degree of stability. These findings further strengthen the conclusion that ACB and BUT are stable inside the pocket, indicating their potential as effective inhibitors.

BUT exhibited remarkable binding characteristics throughout the simulation. At the initial stage (0 ns), BUT formed extensive hydrophobic interactions within the active site and also formed two hydrogen bonds with Gln603 and Asp203, respectively. As the simulation progressed, conformational changes in the active site in response to the binding of BUT led to the formation of new hydrogen bonds with Gly604, Glu300, as well as the catalytic nucleophile Asp443 (Supporting Information S6). Such dynamic adaptation of the active site is critical for maintaining a strong ligand–enzyme association. In contrast, ASG, despite initially exhibiting extensive hydrophobic interactions and forming one hydrogen bond with Gln603, failed to maintain stability within the active site. The reasons for greater stability of BUT compared to ASG in the active site are (i) additional hydrogen bonds formed with Gly604, Glu300, and Asp443; (ii) hydrophobic interaction with Phe575, an active site residue crucial for enzyme–inhibitor stability;^54^ and (iii) greater stability in the inserted loop region preventing widening of the active site and retention of the ligand. These observations suggest that the nature of binding BUT is characterized by a fine balance of hydrophobic and hydrogen bonding interactions, coupled with its ability to stabilize key structural elements of NtMGAM, making it a potential NtMGAM inhibitor. This is reinforced further by its ability to make adaptations during binding interactions as well as to remain stable within the active site pocket over time. Findings of all these studies (RMSDs, RMSF, close contacts analysis, H bonding analysis, and Ligplot analysis) were found to be consistent.

#### 3.2.4 MMPBSA Analysis

**Figure 12:**
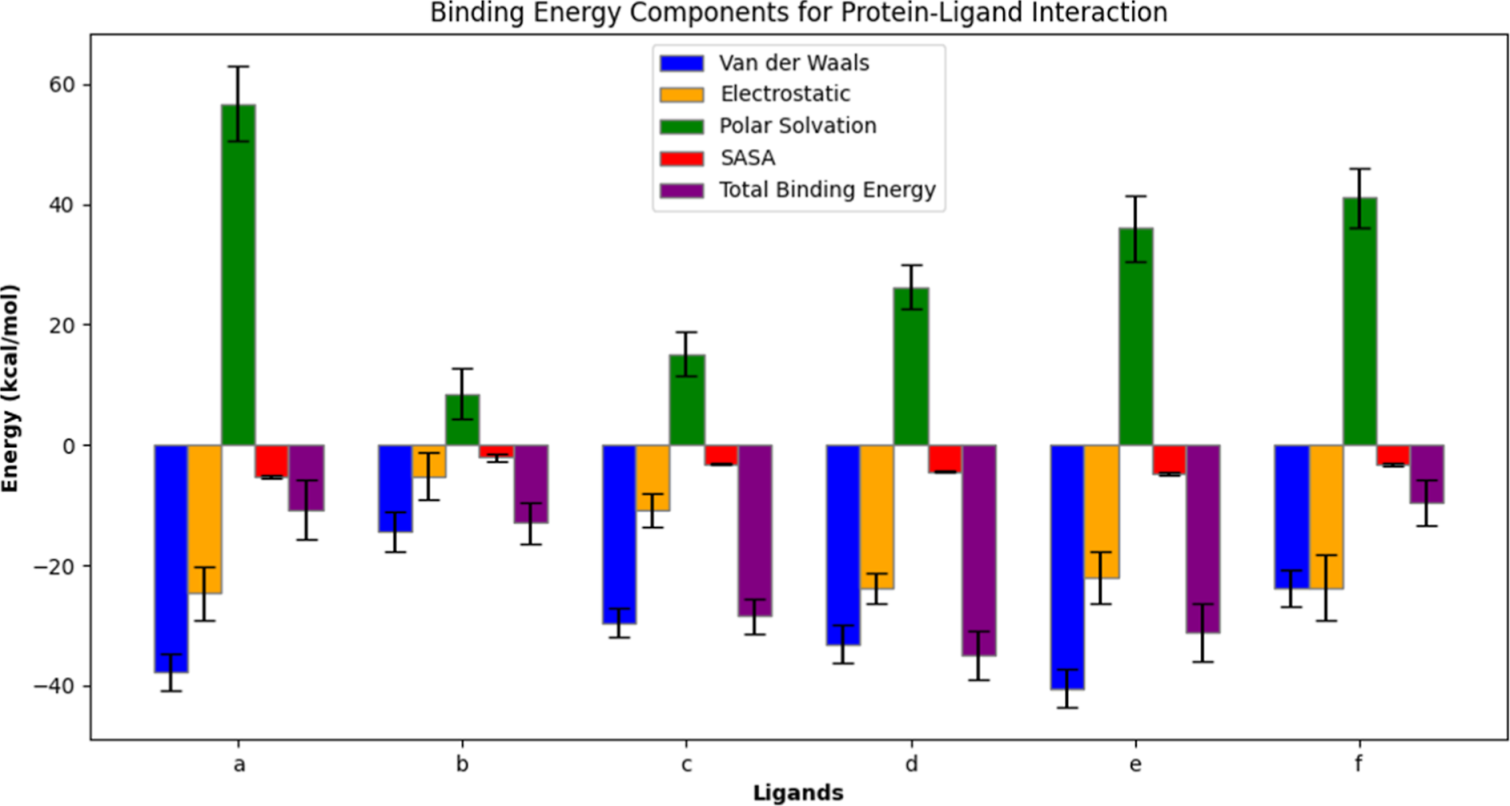
MM-PBSA results for protein–ligand complexes over the last 10 ns frame. Each subplot represents a different ligand: (a) ACB, (b) BEN, (c) BOT, (d) BUT, (e) ALD, and (f) ASG. BUT has the maximum binding energy followed by ALD. BOT also exhibited good total binding energy due to the low polar solvation energy even though the electrostatic energy is less. ACB had very less binding energy even though it had good electrostatic and van der Waals energy due to the high polar solvation energy.

While Ligplot + analysis highlights protein–ligand interaction profiles, MM-PBSA analysis provides a quantitative measure of binding energy in their interactions, reflecting a balance between favorable (van der Waals and electrostatic) and nonfavorable (polar solvation) components (Figure 12). MM-PBSA analysis reveals that among all the six molecules, BUT had the strongest binding energy at −35.01 kcal/mol. It also exhibited strong electrostatic force (−23.77 ± 2.59 kcal/mol) as well as van der Waals energy (−33.06 ± 3.22 kcal/mol). It is followed closely by ALD (−31.13 kcal/mol) and BOT (−28.46 kcal/mol). These results were comparable with those obtained for inhibitors like DSK (−41.90 ± 1.14 kcal/mol) and Luteolin (−18.1401 kcal/mol).^54,58^ BEN, ASG, and ACB showed weaker affinities comparatively (Table 2). Despite showing strong polar and nonpolar interactions with the protein complex, ACB had a higher polar solvation energy, which resulted in it exhibiting a lower favorable total binding energy (−10.81 ± 4.92 kcal/mol). Similar results were observed in the study conducted by Zhang et al.^54^ This underscores the importance of considering ligand–protein interaction profiles along with their binding energetics for understanding and determining the potential of a naturally occurring metabolite to be considered as an effective AGI. BUT, with robust van der Waals and electrostatic energy and lower polar solvation energies, exhibited highest total binding energy which correlates with an experimental IC50 value of 16 μM.^61^

**Table 2:** Details of the Binding Energy (MM-PBSA) and the Physicochemical Properties of the Selected 5 PCs

		physicochemical properties			
LIGS	binding energy (kcal/mol)	MW	TPSA	nHet	flex
BOT	–28.46	378.11	96.22	6	0.08
BEN	–12.99	324.19	86.99	5	1.57
BUT	–35.01	454.16	122.52	8	0.40
ASG	–9.52	372.12	117.2	7	0.21
ALD	–31.13	458.16	153.75	9	0.42
ACB	–10.81	645.25	321.17	19	0.38

MM-PBSA analysis further supported the results of correlation studies carried out between the physicochemical properties of the 45 PCs and their respective binding energies (Table 2). As expected, BUT and ALD with significantly higher binding energy values compared to BOT, BEN, and ASG also possessed higher MW, TPSA, and nHet.

Even though ACB possessed high favorable (electrostatic and van der Waals) binding energy, it also exhibited, on the other hand, high unfavorable (polar solvation) binding energy, which could be due to its very large TPSA. This ultimately resulted in its overall low binding energy. Stringent guidelines are followed when selecting molecules for clinical trials. Among the properties that contribute to drug likeness properties, MW and TPSA are very critical and play a significant role in designing drugs.^62^

**Figure 13:**
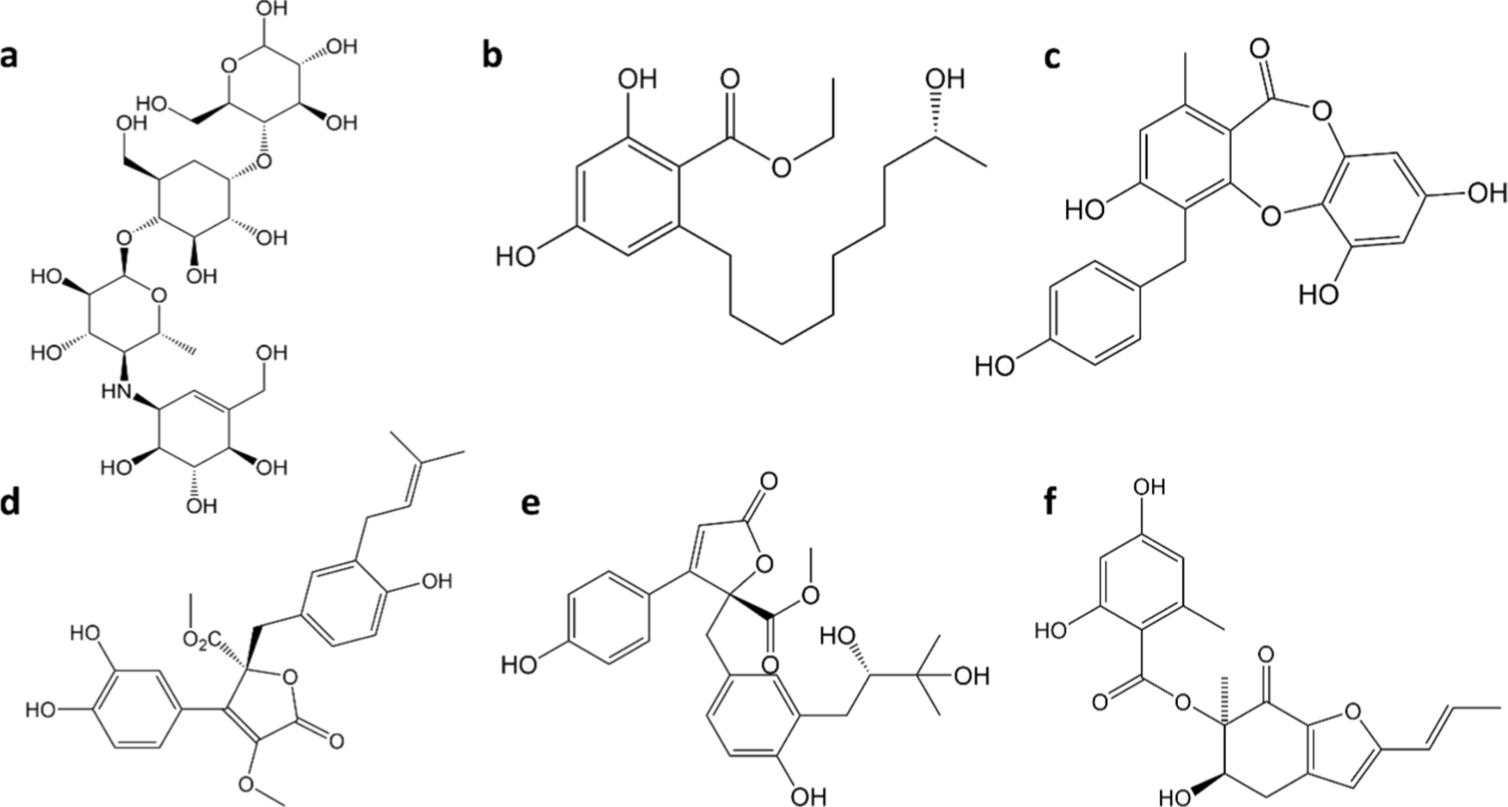
Molecular Structure of (a) ACB, (b) BEN, (c) BOT, (d) BUT, (e) ALD, and (f) ASG.

BUT is a unique molecule with a butenolide core bound to a catechol unit and a prenylphenol. The prenyl group in BUT could have the advantage of having lower polar solvation energy compared to ALD (Figure 13). Among all PCs analyzed using ProTox 3.0, BUT stood out as one of the least toxic molecules. RMSF, close contact, H bonding interaction, Ligplot analysis, as well as MM-PBSA analyses, along with significantly low IC50 value (16 μM)^61^ clearly indicate that among the numerous PCs produced by EF, BUT has the maximum potential of being an efficient and safe AGI and deserves to be evaluated further through in vivo studies.

## 4 Conclusions

T2D poses a significant global health challenge, necessitating the development of effective therapeutic strategies. AGIs have emerged as a potent class of therapeutic agents for managing T2D. EF represents an underexplored source of novel PCs that not only exhibit significant α-glucosidase inhibition activity but also produce minimal side effects. To understand the binding dynamics of PCs with NtMGAM, five ligands belonging to five different classes (depsidones, phenolic acids, butenolides, furanones, and polyketides) were selected for MD simulations. BUT and ALD, with butenolide scaffolds, emerged as the molecules with the highest binding energies (−35.01 and −31.13 kcal/mol, respectively), the maximum number of close contacts, and hydrogen bonds with the least toxicity among the PCs analyzed. Ligplot analysis revealed that while BUT was bound deep inside the active site, ALD interacted primarily with the inserted loop of the active site. The key observation was that BUT-induced conformational changes in the active site brought about greater stability of the ligand within the pocket. In addition, BUT demonstrated a high affinity for key residues Asp443 and Phe575. Interactions of BUT with bulky hydrophobic residues Trp406 (inset 1) and Phe450 (inset 2) lining the active site further enhanced its stability in the pocket. Our findings provide valuable insights into the complexities of protein–ligand interactions, paving the way for designing molecules for therapeutic use. We conclude that PCs with butenolide scaffolds such as BUT and ALD are excellent AGIs with great potential in the treatment of T2D. However, the potential of these butenolide derivatives for therapeutic use requires further validation through in vivo experimentation.

## Supporting Information Available

Details of IC_50_ values, DLSs, and toxicity levels for 150 PCs from EF-S1, details of binding energies, physicochemical properties and toxicity profiles for the 45 selected PCs from EF-S2–5, and details of ligand–protein Interactions of the best 5 ligands at 0 and 300 ns-S6 (XLSX)
